# Wettability of Biofouled Submerged Substrata: Exploratory Relationships with Freshwater Environmental Characteristics

**DOI:** 10.3390/ma19194231

**Published:** 2026-10-06

**Authors:** Katarzyna Boniewicz-Szmyt, Stanislaw Chelkowski, Krzysztof Dorywalski, Pawel Rochowski, Stanislaw Pogorzelski

**Affiliations:** 1 Department of Physics, Gdynia Maritime University, Morska 81-87, 81-225 Gdynia, Poland; k.boniewicz@wm.umg.edu.pl; 2 Institute of Experimental Physics, Faculty of Mathematics, Physics and Informatics, University of Gdańsk, Wita Stwosza 57, 80-308 Gdansk, Poland; stanislaw.chelkowski@ug.edu.pl (S.C.); krzysztof.dorywalski@ug.edu.pl (K.D.); stanislaw.pogorzelski@ug.edu.pl (S.P.)

**Keywords:** freshwater biofouling, trophic status indexes, submerged solids, surface wettability, contact angle hysteresis, bioassessment indicators

## Abstract

This study investigates the wetting behavior of artificial substrates: polymers, metals, and glass, and natural freshwater-submerged substrata represented by *Potamogeton lucens* leaves, collected from shallow eutrophic lakes in northern Poland. Surface interactions were quantified by contact angle measurements and interpreted using the dynamic contact angle hysteresis formalism, with particular emphasis on the apparent surface free energy *γ_SV_*, its polar and dispersive components, adhesive spreading pressure *Π*, work of adhesion *W_A_*, and spreading *W_S_*. These parameters characterize substrate-specific surface differences associated with aging and biofouling. Contact angles were measured using a previously developed captive bubble system, enabling the characterization of fully hydrated, heterogeneous submerged surfaces. Potential relationships between selected lake water characteristics and macrophyte surface wettability parameters were explored using Spearman’s rank correlation. Measurements of young and old leaves from three lakes revealed consistent age-associated changes in advancing contact angle and apparent surface energetic parameters. Exploratory analysis revealed potential relationships between the advancing contact angle and Secchi depth in both leaf-age groups, as well as between age-associated changes in wettability and ammonium concentration or conductivity. The results suggest that wettability analysis may help characterize submerged surface conditions and identify environmental relationships that warrant further investigation.

## 1. Introduction

Rocks and other hard surfaces are generally preferred substrates for biofouling studies. Where these are unavailable, artificial substrates provide a practical alternative. Natural substrates such as macrophytes and macroalgae may also be suitable under specific environmental or experimental conditions [1]. Wettability, commonly characterized using contact angle (CA)-derived parameters, is closely related to the adhesive properties of solid surfaces. The strength of adhesion at solid–water interfaces influences a range of processes, including the transport and redistribution of the active sediment layer, the colonization of benthic substrates and submerged macrophytes by aquatic organisms, and the attachment or release of biofouling. Biofouling development in aquatic environments generally proceeds through three successive stages: formation of a conditioning film, microfouling, and macrofouling. The conditioning film develops rapidly, typically within seconds to minutes of immersion, and forms an interfacial layer that mediates subsequent interactions between fouling organisms and the substrate surface [2]. This process is primarily governed by physical interactions, including Brownian motion, electrostatic forces, and van der Waals interactions. The structure and composition of the resulting conditioning film depend on both water chemistry and substrate properties. Its formation is typically initiated by the adsorption of organic molecules and dissolved ions onto the submerged surface [3]. Microorganisms attached to submerged surfaces secrete extracellular polymeric substances (EPSs), including proteins, glycoproteins, glycolipids, extracellular DNA, and polysaccharides. These compounds form a hydrated, heterogeneous matrix that promotes microbial cohesion and anchors the developing biofilm to the substrate [3]. Recent studies have further demonstrated that substrate wettability, surface topography, and the physicochemical properties of colonizing microorganisms jointly influence microbial attachment and subsequent biofilm development [4,5]. The outermost surface of submerged substrates can be characterized using the captive bubble technique previously described in [6], which enables measurements of surface wettability at different stages of biofilm development, from initial conditioning to more advanced fouling. The captive bubble method, in combination with the axisymmetric bubble shape analysis-profile (ABSA-P), stands for the most practical technique to be applied to water-submerged solid surfaces of porous, fully hydrated, and heterogeneous nature, where capillary penetration and high spreading do not permit the use of the sessile drop method [7]. Wetting measurements are among the simplest and most widely used techniques for surface characterization. At the same time, they are exceptionally surface-sensitive, probing only the outermost approximately 0.5–1 nm of the material [8].

Several well-established methods are commonly used to determine the surface free energy of solids and its individual components from contact angle measurements [9]. The contact angle hysteresis (CAH) approach, developed by Chibowski in [10], relates the total apparent surface free energy of the solid (*γ_SV_*) to three observables: the surface tension of the probe liquid *γ_LV_*, and two wetting angles (CA): the advancing *θ_A_* and receding *θ_R_*, with CAH = *θ_A_* − *θ_R_*. The approach leads to a broad set of surface characteristics, including: the apparent surface free energy *γ_SV_*, adhesive layer film pressure *Π*, work of adhesion *W_A_*, and spreading *W_S_*. All these properties were also determined here to quantify the wetting properties of model artificial substrata (bare and covered glass plates, polymers, metals, etc.), and natural substrata (aquatic macrophytes, in particular *Potamogeton lucens*). Since aquatic macrophytes can act as bioindicators of water chemistry, their surface wettability may reflect plant surface responses to aging and biofouling, further related to trophic state indicators, as explored in model studies performed in four shallow, dimictic, and eutrophic lakes located in the Upper Radunia drainage basin (Kashubian Landscape Park, northern Poland). Previous studies established the applicability of contact angle measurements and the CAH approach to mineral surfaces exposed to natural seawater surfactants, extended the captive bubble methodology to submerged artificial and natural substrata, including aquatic macrophytes [11], and combined wettability measurements with complementary techniques for monitoring marine biofilm development and examining relationships with water-quality parameters [6]. Building on this methodological background, the present study focuses on freshwater lake environments, with particular attention to the wettability characteristics of artificial sampling substrata, biofilm accumulation, and the influence of sampler configuration on the observed results.

To date, the wetting behavior of solid mineral surfaces in surfactant-containing seawater has been investigated under laboratory conditions using a broad range of well-characterized stone samples with varying hydrophobicity. These studies employed sessile-drop, captive-bubble, and inclined-plate techniques, together with contact angle hysteresis analysis [11,12]. As shown recently, surface wettability changes induced by successive application of reactive mouthrinses and recovering cleanser liquids to dental polymer (PMMA), as well as paint layer covering of metals, can also be expressed quantitatively via corresponding variations in several surface parameters [13,14,15,16].

Building on these earlier methodological developments, the present study focuses on the comparative characterization of wettability changes in artificial and natural freshwater substrata under environmental exposure, with particular attention to biofouling-related changes and their relationships with selected water-quality parameters.

The aim of the paper is threefold: (1) to apply the established CAH-based methodology and computer-operated captive bubble system to characterize the wettability of artificial and natural substrata collected from freshwater environments, and to examine its potential for assessing surface changes associated with biofouling; (2) to select the optimal collecting substrata, on the basis of their wettability signatures, in biofouling studies; and (3) to explore potential relationships between trophic-state-related lake water characteristics and surface wettability parameters of submerged substrata, with particular attention to relationships warranting further investigation in the context of freshwater bioassessment.

## 2. Liquid/Solid Wettability—Key Relations

The classical force balance at the three-phase contact line, expressed by the Young’s equation—Equation (1), relates the equilibrium contact angle, *θ_eq_*, to the interfacial free energies of the three interfaces involved in the system [17]:

(1) $$\gamma_{\mathrm{SV}}^{\left(Y\right)}-\gamma_{\mathrm{SL}}=\gamma_{\mathrm{LV}}\cos\theta_{\mathrm{eq}}$$

where $\gamma_{\mathrm{SV}}^{\left(Y\right)}$, *γ_SL_*, and *γ_LV_* are the interfacial free energies of the solid/vapor, solid/liquid, and liquid/vapor interfaces, respectively. The superscript (*Y*) distinguishes the thermodynamic quantity appearing in Young’s equation from the apparent surface free energy evaluated below. Young’s equation describes equilibrium wetting and relates the contact angle *θ_eq_* to the difference $\gamma_{\mathrm{SV}}^{\left(Y\right)}-\gamma_{\mathrm{SL}}$; the individual solid interfacial free energies cannot be independently determined from contact angle measurements [18].

In practice, the equilibrium contact angle is difficult to determine, since the contact line may remain trapped in metastable states. Consequently, advancing (*θ_A_*) and receding (*θ_R_*) contact angles are observed, and their difference defines contact angle hysteresis (CAH = *θ_A_ − θ_R_*). Importantly, CAH may occur even on macroscopically smooth and chemically homogeneous surfaces, owing to effects operating at microscopic and molecular length scales [18,19].

Contact angle hysteresis may result from surface roughness, chemical heterogeneity, contact-line pinning, and surface adaptation or deformation. Recent theoretical studies have further demonstrated that microscopic interfacial interactions may give rise to distinct energy minima and non-zero CAH even in the absence of macroscopic roughness [18,19]. The influence of surface roughness on the apparent contact angle can be described, under the assumptions of the Wenzel model, by the following relation [20]:

*cosθ_W_* = *rcosθ_eq_* (2)

where *r* is the ratio of the actual to the projected surface area, *θ_W_* is the apparent Wenzel contact angle, and *θ_eq_* is the equilibrium Young contact angle of the corresponding smooth surface. For a chemically heterogeneous surface consisting of two distinct regions, the apparent contact angle can be described by the Cassie–Baxter relation [21]:

*cosθ_C_* = *F*_1_*cosθ*_1_ + *F*_2_*cosθ*_2_ (3)

where *θ_C_* is the apparent Cassie–Baxter contact angle, *F*_1_ and *F*_2_ are the surface fractions of the two regions (*F*_1_ + *F*_2_ = 1), and *θ*_1_ and *θ*_2_ are their respective contact angles. These relations describe the effects of surface structure and heterogeneity on apparent wettability but do not, by themselves, identify the mechanisms responsible for CAH.

In kinetic hysteresis, the observed CAs evolve in time depending on a characteristic system time scale comparable to the time of experiment [22]. According to the CAH approach proposed by Chibowski [10], the receding contact line leaves behind a thin film of the probe liquid, which modifies the surface free energy. In the following, *γ_SV_* denotes the apparent surface free energy of the investigated surface, distinguished from the thermodynamic reference quantity $\gamma_{\mathrm{SV}}^{\left(Y\right)}$ introduced in Equation (1). The apparent surface free energies corresponding to the initial and film-covered states, denoted by *γ_SV_* and *γ_SF_*, respectively, are related as follows:

*γ_SF_* = *γ_SV_* + *Π* (4)

*Π* = *γ_LV_*(*cosθ_R_* − *cosθ_A_*) (5)

where *Π* is the film pressure associated with the liquid film remaining after the contact line has receded. It should be noted that this film originates from the probe liquid and should not be confused with the biofilm formed during substrate immersion in the lake.

The apparent total surface free energy of the investigated solid, *γ_SV_*, can be evaluated from three observables: *γ_LV_*, *θ_A_*, and *θ_R_*, using the CAH approach [10]:

(6) $$\gamma_{\mathrm{SV}}=\frac{\Pi {\left(1+\cos\theta_{A}\right)}^{2}}{{\left(1+\cos\theta_{R}\right)}^{2}-{\left(1+\cos\theta_{A}\right)}^{2}}$$

As emphasized by Chibowski [10], the surface free energy determined from contact angle measurements is an apparent quantity that depends on the interactions between the solid and the probe liquid. This distinction is particularly important for the rough and heterogeneous biofouled surfaces investigated here. Therefore, the calculated energetic parameters are treated as apparent, model-dependent descriptors of surface wettability rather than intrinsic thermodynamic properties.

The work of spreading *W_S_* of liquid on a solid surface is related to the work of adhesion *W_A_*, and the work of cohesion *W_C_* [17]:

*W_S_* = *W_A_* − *W_C_* (7a)

*W_A_* = *γ_LV_*(1 + *cosθ_A_*) (7b)

*W_C_* = 2 *γ_LV_* (7c)

*W_S_* represents a quantity relating the wettability to the mechanical strength of adhesion. It allows one to quantify the competition between solid–liquid adhesions with liquids of differentiated polarity [7].

For non-hysteretic liquid drop/solid systems (CAH = 0), *γ_SV_* can be derived from static CA measurements (assuming *θ_eq_* = *θ_A_*), and thus is expressed with *W_A_*:

(8) $$\gamma_{\mathrm{SV}}=\frac{1}{2}\gamma_{\mathrm{LV}}\left(1+\cos\theta_{A}\right)=W_{A}/2$$

In the total solid surface free energy, being a sum: *γ_SV_* = $\gamma_{\mathrm{SV}}^{p}$ + $\gamma_{\mathrm{SV}}^{d}$ of both polar and dispersive components, the dispersive term $\gamma_{\mathrm{SV}}^{d}$ equals to [17]:

(9) $$\gamma_{\mathrm{SV}}^{d}=\gamma_{\mathrm{LV}}{\left(1+\cos\theta_{A}\right)}^{2}/4$$

## 3. Materials and Methods

### 3.1. Study Sites and Trophic State Indexes

**Figure 1:**
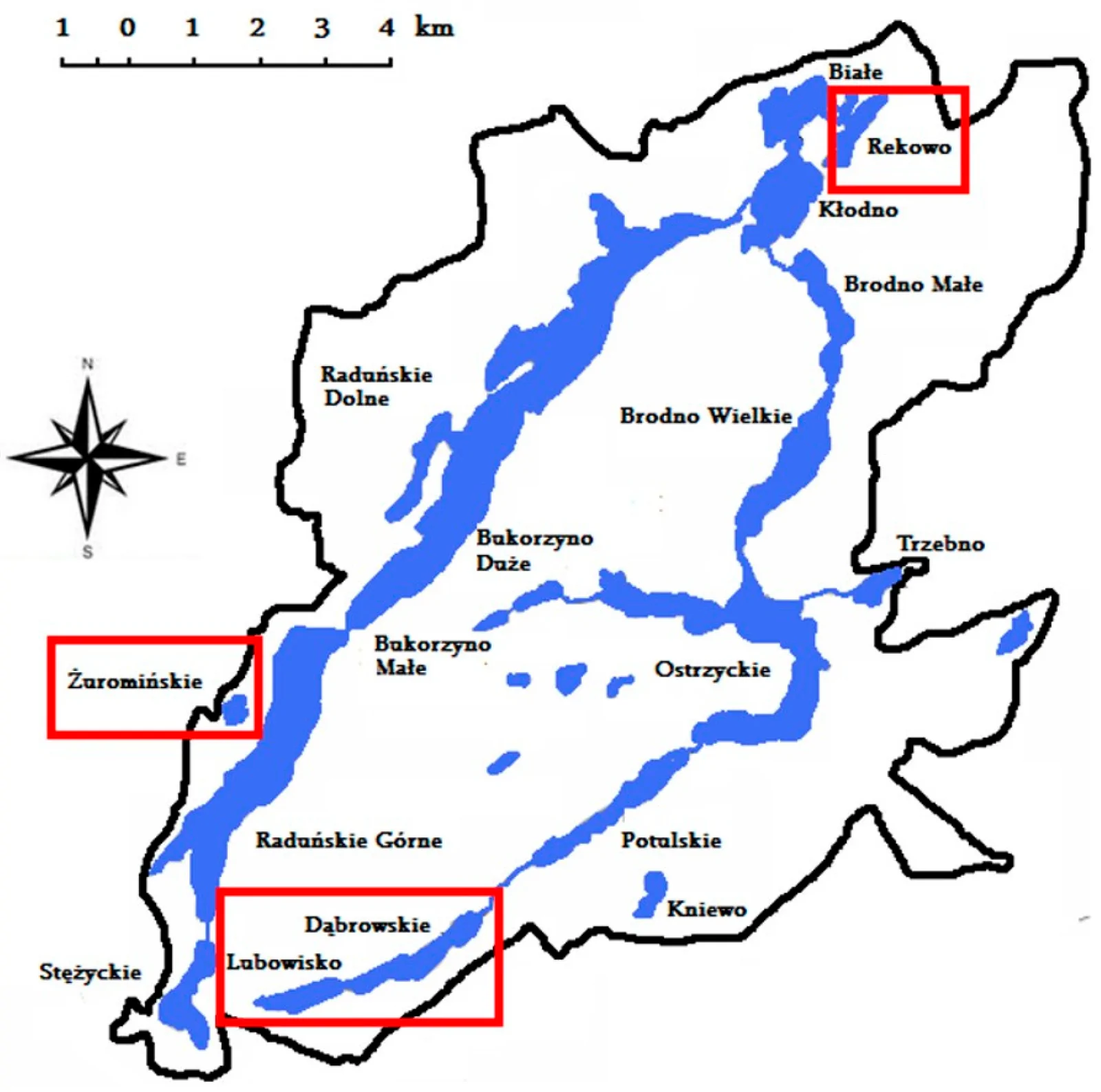
Location of the four investigated lakes (Rekowo, Lubowisko, Dabrowskie, and Zurominskie) within the Upper Radunia drainage basin. The studied lakes are highlighted.

Lakes (Rekowo, Lubowisko, Dabrowskie, and Zurominskie) are located in the Upper Radunia drainage basin (Figure 1; Kashubian Landscape Park, Kashubian Lake District, northern Poland). The Kashubian Lake District is an early post-glacial landscape with a natural environment.

The area of the Kashubian Landscape Park measures 33,202 hectares. The lake density is 10.47%. The studied lakes are reservoirs of glacial origin. The water basin of these lakes is a forest–agriculture area. These lakes are dimictic, with circulation occurring in spring and autumn. Their thermal structure is associated with a moderate type of climate. The nutrient content in the lakes shows seasonal variations associated with biological activity. The physicochemical characteristics of the investigated lakes included pH, dissolved O_2_, nutrient concentrations, conductivity, and Secchi depth (Table 1). Lake trophic status was characterized using Carlson’s trophic state indices calculated from Secchi depth [TSI(SD)], chlorophyll a [TSI(Chl), concentrations were determined spectrophotometrically], and total phosphorus [TSI(Ptot)] [23], with Secchi depth and chlorophyll a concentration determined during a single sampling campaign in August 2024. These parameters provided the environmental reference data for further discussion. Relationships between lake water characteristics and the wettability parameters of *Potamogeton lucens* leaves were explored using Spearman’s rank correlation coefficient. The analysis included three independent lakes, with young and old leaves considered separately. Given the limited number of independent lakes (*n* = 3), Spearman’s rank coefficients were used solely as descriptive measures of the ordering of observations. No inferential interpretation or statistical significance was assigned to these relationships.

### 3.2. Biofilm Samplers

**Figure 2:**
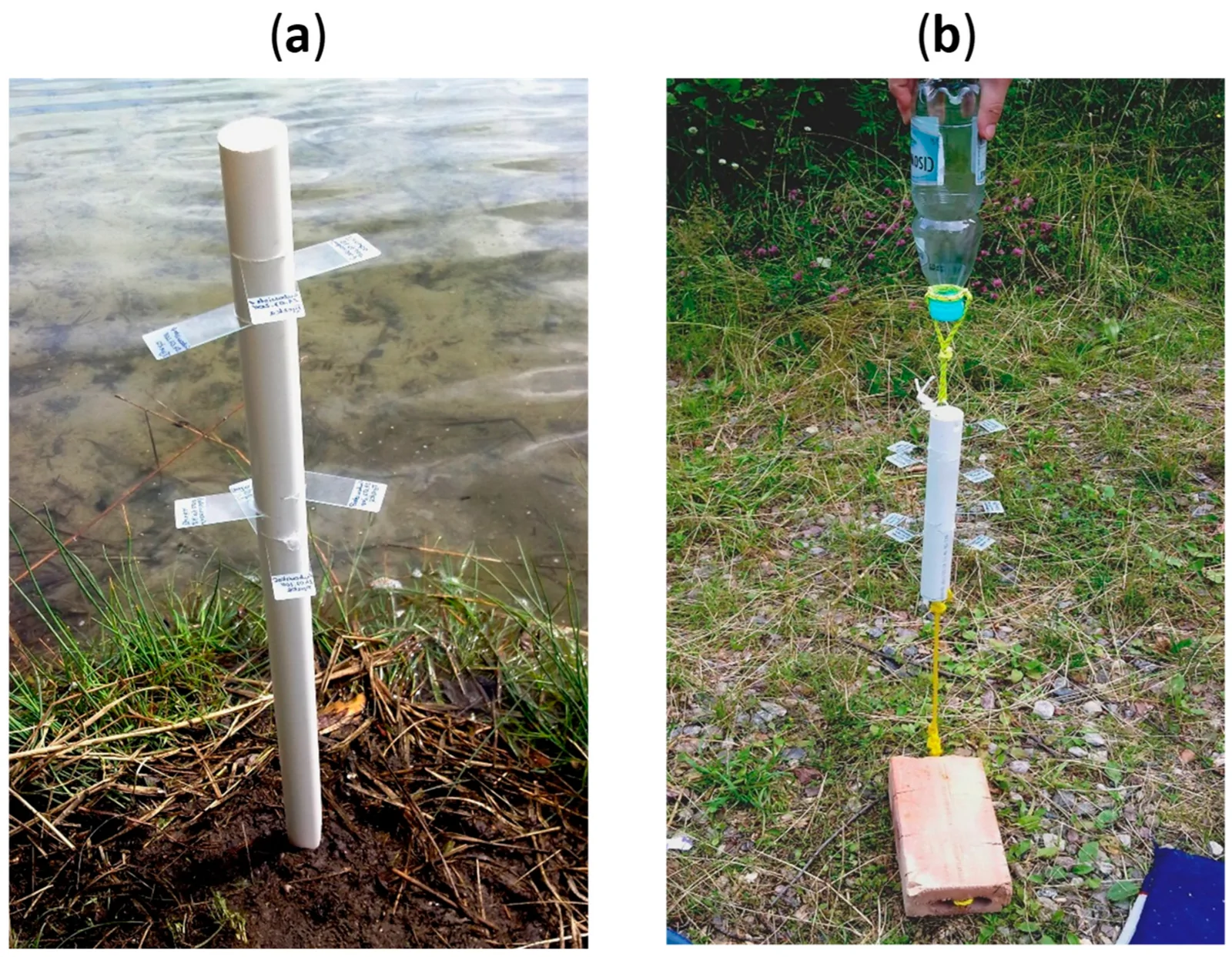
Biofilm plate sampling holders: stick-like (**a**) and float-type (**b**) devices.

A wide variety of solid materials were used as artificial biofilm collectors with differentiated hydrophobicity in the present study, as named and listed in Table 2. They were cut into thin rectangular plates 0.3–2 mm thick, 2–3 cm wide, and 5–8 cm long. The collecting substrata were mounted on two types of supporting devices, stick-type and float-type, as shown in Figure 2a and Figure 2b, respectively, and then submerged in the studied water body at a depth of 0.5 m. The former type of deposition plate holder, which was situated horizontally, was fixed firmly to the bottom, whereas the latter was allowed to float freely. The glass plates consisted of Duran glass from Schott GmbH, Mainz, Germany, and measured 77 × 26 × 1.5 mm. Because the cleaning procedure can markedly affect the measured contact angles, the model substrates were thoroughly cleaned prior to use. The plates were first immersed in a methanol/chloroform mixture (1:2, *v*/*v*), after which the solvent was allowed to evaporate. The same procedure was then repeated using toluene.

The submerged macrophyte (*Potamogeton lucens*) was used as the natural model substrate. During retrieval, the macrophyte leaves were rinsed with water from the respective lake. No additional cleaning or surface preparation was performed prior to contact angle measurements. As a natural material, submerged macrophyte leaves exhibit large structural variability, which is reflected in a significant scatter of the results for any measured property. This difficulty can be minimized by collecting a large number of replicate experimental results (10–15 for each sample here). Rectangular sections of macrophyte leaves (5 × 3 cm) were excised with a scalpel, stretched over a PTFE plate mounted on a horizontal stainless-steel support, and secured at both ends with magnets for contact angle measurements. For each sampling site, 5–10 biofilm samples were collected together with adjacent lake water, transferred to plastic bottles, protected from desiccation, and processed in the laboratory within 1 h of collection.

In total, seven sampling campaigns were conducted in each lake between March and December 2024, at approximately 3–7-week intervals.

### 3.3. Water Phase and Bioaccumulation Characterization

The surface tension *γ_LV_* of the lake water was determined in situ using a Bubble Pressure Tensiometer BP2100 (PocketDyne, Krüss, Hamburg, Germany). The measured values ranged from 71.1 to 73.4 mN m^−1^ and were used to characterize variations in the physicochemical properties of the lake water and as a liquid surface tension probe for the characterization of the wetting properties of submerged samples. The surface roughness (SR) of solid biofouled substrata was analyzed using confocal laser scanning microscopy (Axiovert 200M, Carl Zeiss, Oberkochen, Germany). This allowed the calculation of the arithmetic mean SR from a mean plane within the sampling area (245 × 254 × 60 μm). Moreover, the biofilm surface architecture images revealed additional structural details quantified in terms of mean thickness, biovolume, coverage area fraction, fractal dimension, Hopkins aggregation index, etc., as demonstrated in [24]. Bioaccumulation of the samples over time was quantified using the biofilm wet mass (BWM). At each sampling site, the sampling substrata were scraped with a cutter blade from the known area, and the biofilm wet mass (mg cm^−2^) was evaluated with an electronic balance. No visible damage to the substrates was observed during scraping. Nevertheless, incomplete biofilm removal cannot be entirely excluded and may lead to an underestimation of the total accumulated biomass. Therefore, BWM was treated as the wet mass of the recovered material rather than an absolute measure of biofilm accumulation.

### 3.4. Surface Wettability Measurements

The surface tension of the reference probe water was measured before each experimental session using the BP2100 tensiometer. Since the water originated from the same source and the measurements were performed under identical thermal conditions, the small variation in the measured surface tension was attributed primarily to the repeatability of the measurement procedure. For the measurements presented in Table 2, the mean surface tension and its standard deviation (*γ_LV_* = 71.9 ± 0.7 mN m^−1^) were used to calculate the wettability parameters and estimate their uncertainties. The measurements of artificial substrata presented in Table 3 were performed in a double experimental session. The surface tension of the probe water (natural Rekowo Lake) differed by only 0.1 mN m^−1^ between the two sampling dates, which was comparable to the stated measurement accuracy using reference probe water with almost same surface tension of *γ_LV_* = 73.4 mN m^−1^. For the macrophyte leaf measurements presented in Table 3, the surface tension of the probe water was 71.5 mN m^−1^ for Dabrowskie Lake and 72.8 mN m^−1^ for Lubowisko and Zurominskie lakes.

**Figure 3:**
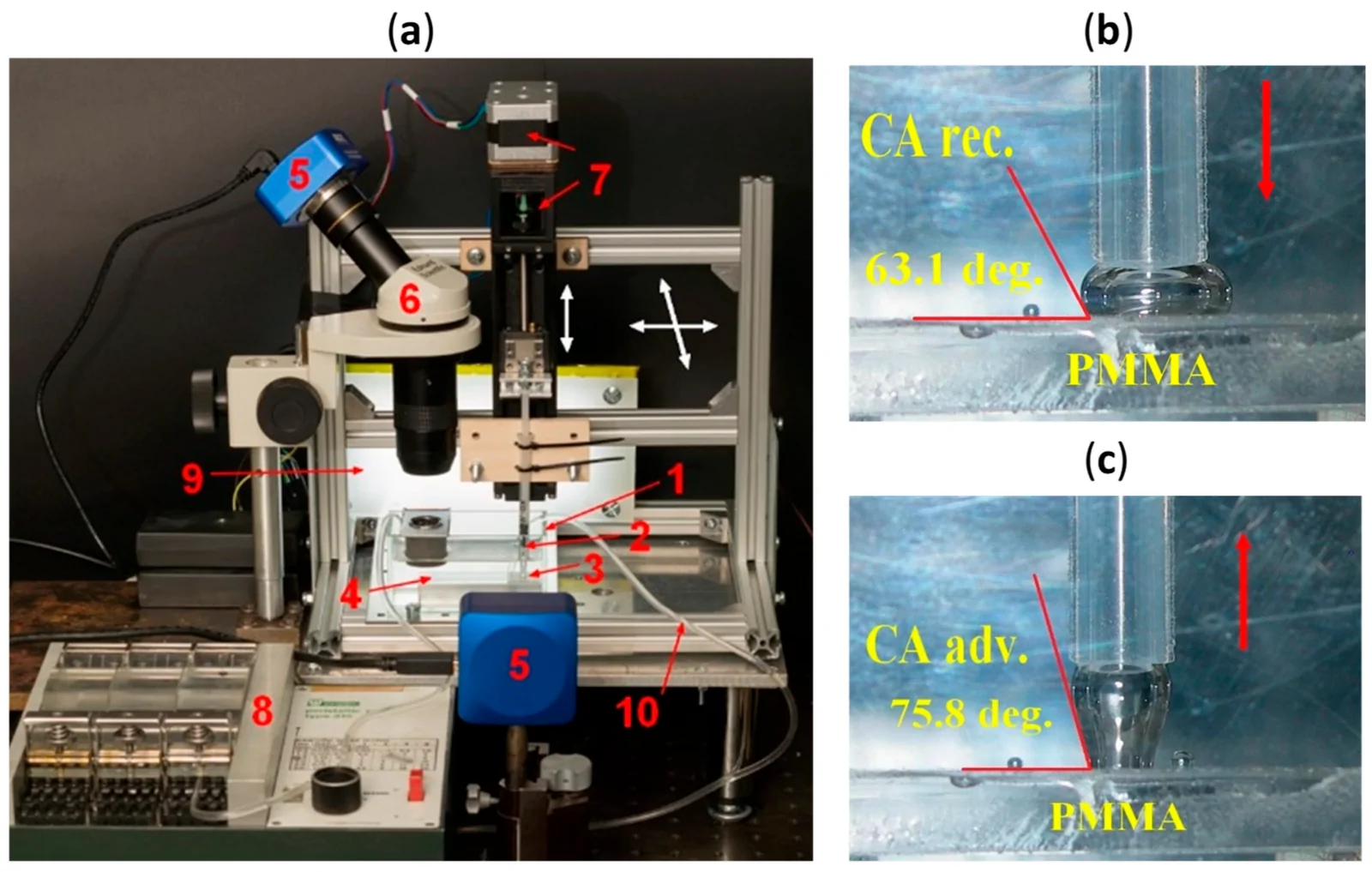
(**a**) Automated, computer-operated captive bubble setup, for dynamic CAs measurements (adapted from [6]); determination of the receding *θ_R_* (**b**), and advancing *θ_A_* (**c**) contact angles for polymer (PMMA)/water system. The red arrows indicate the direction of the syringe movement.

The computer-operated captive bubble system used for CAH measurements is presented in Figure 3a. The experimental setup, its characteristics, and the measurement procedure have been described previously in [6]. Briefly, a bubble was formed at the tip of a 25 μL microsyringe and maintained in the water phase for a predefined period before being brought into contact with the investigated surface. The bubble was then confined between the syringe tip and the substrate and compressed using a precision micrometric stage. The receding contact angle, *θ_R_*, was determined from the side-view images, as illustrated in Figure 3b. The procedure was subsequently repeated with a bubble of the same volume while reversing the motion of the vertical micrometer stage. As the bubble was withdrawn upward (Figure 3c), the advancing, i.e., maximum, contact angle was determined. The acquired photographs were analyzed with graphical software (ImageJ 1.41, National Institute of Health, Bethesda, MD, USA) to determine both the dynamic contact angles, and the fraction of the total surface covered by irregular regions of settled organic matter. For each studied surface, 4 to 8 measurements were performed at different testing spots on the substrate to evaluate spatial homogeneity. The individual CA measurement error was within 1°, and the repeatability of each measurement was approximately ±2°. The uncertainties of the derived wettability parameters were estimated by first-order uncertainty propagation, assuming an uncertainty of ±1° for each contact angle and a probe liquid surface tension uncertainty of ±0.2 mN m^−1^, unless otherwise specified. The captive bubble method, in conjunction with the axisymmetric bubble shape analysis-profile (ABSA-P) procedure, allowed reproducible measurements of contact angles on porous mineral surfaces.

It should be pointed out that the measurement of dynamic contact angles is dependent on contact line motion at high capillary numbers. The capillary number represents the ratio of viscous to capillary effects and is defined as *Ca = μV/γ_LV_*, where *μ* is the liquid viscosity and *V* is the characteristic liquid front velocity. Dynamic contact angles are independent of contact line motion at low capillary numbers (*Ca* < 10^−5^) [8].

## 4. Results

### 4.1. Sampling Substrata Selection

As a first step, the wettability characteristics of a broad range of artificial substrata were determined using similar aqueous probe liquid to provide comparative data for the selection of biofilm samplers. The investigated materials included polymers, metals, and composite surfaces with different wetting properties. Previously published measurements of bare glass and *Potamogeton lucens* leaves [11] were also included in Table 2 as additional literature reference data. The use of a common probe liquid for the investigated artificial materials enables a direct comparison of their contact angles and CA-derived energetic parameters under consistent liquid-phase conditions.

The effect of surface energy on adhesion is often studied by employing a wide range of materials, from urethanes and epoxies (high energy) to silicones and fluorinated materials (low energy) [25]. A general observation is that hydrophobic surfaces tend to adsorb proteins from solutions, whereas hydrophilic surfaces are more resistant. Baier published results indicating that the amount of bioadhesion does not correlate well with the surface energy of the substratum [26]. He reported that there is a surface-energy window (20–30 mJ m^−2^) within which adhesion is minimal. Substrata with surface energies below 20 and above 30 mJ m^−2^ exhibited appreciable amounts of adsorbed biomass. As shown in Table 2, paraffin wax-coated glass, PTFE, PVDF, PSU, PMMA, and the gelatinous layer were tentatively expected to exhibit lower organic matter collection efficiency based on their wettability characteristics. The CAH values within this group varied considerably, indicating that the anticipated differences in organic matter collection efficiency cannot be attributed to CAH alone. Other substrata are hydrophilic, and show much larger values of CAH, *γ_SV_*, and $\gamma_{\mathrm{SV}}^{d}$*/γ_SV_*. The same characteristics were exhibited by the natural substrate (see 16 in Table 2). Low values of CAH and Π are characteristic of hydrophobic materials, whereas the remaining parameters, i.e., *γ_SV_*, *W_A_*, *W_S_*, and $\gamma_{\mathrm{SV}}^{d}$*/γ_SV_*, correspond to hydrophilic substrata (10, 11 in Table 2). Wetting in plants is influenced by cell structure and by fine surface features, such as cuticle folding or epicuticular wax [27]. Permanent wet surfaces occur in submerged aquatic plants, as studied here. Extensive SEM analysis of aquatic plants showed that submerged leaves have relatively smooth cell surfaces (tubular or slightly convex cells) and lack three-dimensional waxes, cell papillae, or hairs [28]. A similarly flat surface, with slight roughness (*r* = 1.02 ± 0.01), was observed in *Potamogeton lucens* leaves (see Figure 2 in [11]). Moreover, in a previous study (see Figure 3 in [11]), the CA (=48.3°) of a heterogeneous glass surface partially covered with filamentous algae allowed the dark-area fraction *f* (=0.44) to be evaluated with the Cassie–Baxter correction model.

An obvious advantage of permanent wetting in submerged aquatic plants is that they experience no drought-induced stress. It has been shown that, for self-cleaning by moving water, the surface CAH should be less than 10° [29], as found here. There is evidence that CAH has some effect on biofouling. Schmidt et al. confirmed that, although the amount of biofouling did not correlate with surface energy of coatings [30], the adhesive properties of the surface correlated with water CAH; coatings with the best fouling-release properties exhibited the lowest CAH. In addition to surface free energy, other factors, including surface charge, surface roughness, temperature, contact time, substratum color, and fluid shear flow velocity, also have a significant influence on biofouling adhesion [31,32]. Surface energy is a combination of dispersion (non-polar) and polar contributions. Dispersion energy exists between all molecules, whereas polar energy exists only when polar groups are present [33]. As a result, the hydrophilic–hydrophobic balance of biofouling organic matter molecules is a key factor to consider when selecting appropriate film samplers.

### 4.2. Biofilm Collection Efficiency Versus Surface Wettability

Artificial substrata are widely used in biofouling studies, where biofilm accumulation and surface wettability provide complementary information on the state of submerged surfaces. A wide variety of artificial substrata have been used in previous studies, including polyurethane foam units, Styrofoam spheres and blocks, plastic Petri dishes, glass coverslips, glass and acrylic slides, Plexiglas disks, glazed and unglazed clay tiles, cut rock surfaces, clay flowerpots, wooden dowels and sticks, plastic tape, and artificial plastic plants. A comprehensive review of these applications is provided in [34]. Evidence suggests that diatom communities colonizing newly introduced substrata respond more rapidly to changes in water quality than mature communities established on natural surfaces [35]. In general, artificial substrata have been applied in freshwater environments. Initial cell attachment to the conditioning film is rapid and occurs over a period of several seconds to minutes [2]. Secondary microfouling colonizers, such as spores of macroalgae and protozoa, attach to the conditioning film on submerged substrates in about a week [36].

The efficacy of different foul-release coatings varies by target species. Hydrophilic surfaces are more effective against protein and cell adhesion, whereas hydrophobic surfaces are more effective against macrofouling [37]. The present study examines biofilm accumulation on artificial samplers during environmental exposure. Biofilm wet mass (BWM) was used to quantify the accumulated material, whereas contact angle measurements characterized the wettability of the exposed surfaces. Table 3 presents results for glass and PVC samplers deployed in Rekowo Lake, together with reference data for *Potamogeton lucens* leaves collected near the Baltic coastal waters [11] and from the investigated lakes.

The bare glass sampler was a highly hydrophilic substrate, with *θ_eq_*, *θ_A_*, *θ_R_*, and CAH values comparable to those exhibited by clean mineral plates. The surface energy parameters indicated mainly hydrophobic liquid/solid interactions, with the dispersive contribution to the surface free energy equal to 86–90% of *γ_SV_*. The accumulation of organic matter on the glass samplers was assessed after exposure periods of 19 and 46 days in freshwater. Compared with the previously published glass reference, the exposed glass surfaces exhibited reduced contact angle hysteresis (CAH), primarily resulting from an increase in the receding contact angle (*θ_R_*). This was accompanied by a decrease in film pressure (*Π*) and an increase in apparent surface free energy (*γ_SV_*), consistent with greater water wettability rather than hydrophobization. However, these differences should be interpreted with caution, as the reference and exposed samples were nominally made of the same material but were measured using different natural aqueous probe liquids (still of comparable *γ_LV_*).

A similar tendency towards greater water wettability was observed for the exposed PVC surfaces, although the changes in contact angles differed from those found for glass. Compared with the reference PVC (Table 2), all exposed PVC samplers exhibited lower advancing and receding contact angles, with *θ_A_* decreasing from 77.4° to 34.9–57.8° and *θ_R_* from 55.9° to 28.4–45.7°. The larger reduction in *θ_A_* resulted in a decrease in CAH from 21.5° to 6.5–12.1°. This was accompanied by a reduction in film pressure (*Π*) from 24.6 to 4.4–12.2 mN m^−1^ and an increase in apparent surface free energy (*γ_SV_*) from 38.4 to 53.4–65.7 mJ m^−2^. The work of adhesion (*W_A_*) also increased from 87.6 to 112.5–133.6 mJ m^−2^. Thus, despite the different responses of the receding contact angle, both glass and PVC exhibited lower CAH and higher apparent surface free energy relative to their respective reference data, consistent with greater water wettability. As for glass, however, differences in the aqueous probe liquids and the absence of matched pre-exposure controls prevent these changes from being attributed exclusively to biofilm formation.

Other pronounced differences in wettability were associated with the sampler configuration and were particularly evident for PVC. For the stick-like samplers, both PVC foils exhibited higher advancing contact angles and lower apparent surface free energies than glass. In contrast, the float-type samplers showed a different relationship: the advancing contact angle of orange PVC was substantially lower than that of glass (34.9° versus 45.7°), whereas its apparent surface free energy was higher (65.7 versus 61.4 mJ m^−2^). A similar, although less pronounced, tendency was observed for brown PVC. For orange PVC, changing from the stick-like to the float-type configuration was also associated with a decrease in CAH from 12.1° to 6.5°. These observations demonstrate that the relative wettability characteristics of glass and PVC depend on the conditions associated with sampler deployment.

A similarly pronounced dependence on sampler configuration was observed for biofilm accumulation. At the first sampling, the float-type samplers exhibited substantially higher BWM than their stick-like counterparts for all three materials. However, the BWM values recorded at the second sampling followed opposite patterns for the two configurations: they were higher for all stick-like samplers and lower for all float-type samplers. Consequently, the stick-like PVC samplers exhibited higher BWM than the corresponding float-type samplers at the second sampling, whereas the opposite relationship persisted for glass. These findings indicate that the relative biofilm collection performance of the two sampler configurations changed over the investigated exposure period and that this behavior was material-dependent. Since BWM was determined at only two exposure times (19 and 46 days), differences between sampling dates cannot be interpreted as direct measurements of biofilm growth or detachment rates, as they may reflect changes in environmental conditions. Nevertheless, comparisons between materials and sampler configurations within each sampling date were performed after the same exposure duration.

The orange PVC samples accumulated more biofilm wet mass than the brown PVC samples in both sampler configurations and at both sampling dates (Table 3). This observation is consistent with our previous findings [24], which revealed enhanced biofilm accumulation on PVC surfaces whose diffuse-reflectance spectra exhibited substantial overlap with the absorption bands of photosynthetic pigments. The earlier study suggested that light reflected and scattered by the substrate may influence the colonization of phototrophic organisms. The present results indicate that a similar color-associated difference in biofilm accumulation can also be observed under freshwater conditions. However, since the reflectance spectra and local illumination conditions were not measured in the present experiment, the proposed optical mechanism cannot be identified as the sole cause of the observed differences.

The wettability signatures of natural substrata (rows seven and eight in Table 3, adopted from [11]) were studied for young, newly grown and 1-year-old leaves. These historical reference data were extended by measurements performed on young and old *Potamogeton lucens* leaves collected from three freshwater lakes: Dabrowskie, Lubowisko, and Zurominskie (rows 9–14, Table 3). The wettability of the exemplary macrophyte surfaces was lower than that of the artificial samplers described above. All CAs were higher, with *θ_A_* = 62.1–73.6° and *θ_R_* = 50.3–65.1°. On the other hand, CAH = 5.0–14.6° and *Π* = 5.5–17.0 mN m^−1^ exhibited considerable variation, whereas *γ_SV_* = 42.8–51.4 mJ m^−2^ and *W_A_* = 92.5–105.8 mJ m^−2^ were generally lower, and *W_S_* = −52.3 to −38.0 mJ m^−2^ was more negative than the corresponding values obtained for the artificial samplers. As shown in [38], CAH is related to adhesion between the liquid and the substratum. In addition, the relative contribution of dispersive interactions to the surface free energy was lower, with $\gamma_{\mathrm{SV}}^{d}$*/γ_SV_* attaining only 0.68–0.77.

The leaf-aging effect was reflected in the following parameter changes. In the previously published measurements [11], *θ_A_* and *θ_R_* decreased by 10° and 14.8°, respectively; CAH and *Π* increased by 4.8° and approximately 4.1 mN m^−1^, respectively; *γ_SV_* and *W_A_* were higher by approximately 11% and 12.6%, respectively; and *W_S_* became less negative (changing from −51.8 to −40.0 mJ m^−2^). Similar trends were observed in the present freshwater study. For old leaves, *θ_A_* and *θ_R_* were lower by 9.9–10.8° and 0.7–13.6°, respectively, while *γ_SV_* and *W_A_* increased by 5.1–8.7 and 11.6–12.5 mJ m^−2^, respectively, and *W_S_* became less negative in all three lakes. However, the changes in CAH and *Π* were not uniform. CAH increased for old leaves from Dabrowskie and Lubowisko lakes, whereas it decreased from 14.6° to 5.0° in Zurominskie Lake. Similarly, *Π* remained nearly unchanged in Dabrowskie Lake (10.2–10.3 mN m^−1^), increased from 9.3 to 12.4 mN m^−1^ in Lubowisko Lake, and decreased from 17 to 5.5 mN m^−1^ in Zurominskie Lake.

The surface energy parameters indicated a substantial contribution of dispersive interactions, i.e., the dispersive term of the surface free energy increased from approximately 0.68 *γ_SV_* to 0.77 *γ_SV_* for old or mature leaves in the historical dataset. A similar increase was observed for the freshwater samples, from 0.679 to 0.699 *γ_SV_* for young leaves to 0.744–0.770 *γ_SV_* for old leaves. In particular, leaf-surface erosion may increase SR and contribute to changes in CAH, while the accumulation of surface-active organic matter may modify *Π* and *γ_SV_* over time. These processes may occur simultaneously, and their individual contributions cannot be distinguished from the present measurements. Thus, the studied leaf surfaces become less hydrophobic with age, although the accompanying changes in CAH and *Π* depend on the investigated lake. Future controlled leafaging experiments under axenic conditions, compared with naturally biofouled leaves, could help distinguish the contributions of intrinsic leaf aging and microbial colonization to the observed changes in wettability.

Overall, the biological leaf-blade surface can be regarded as a solid substratum with distinct surface energetic properties. Following immersion, several processes, including wax erosion, adsorption of naturally occurring surfactants, bacterial and algal colonization, and biofouling, may proceed concurrently, resulting in time-dependent changes in surface characteristics. Consequently, a set of wettability-related parameters may serve as a characteristic signature of macrophyte leaf condition and may further reflect chemical stress or varying environmental conditions in natural waters.

Biofilm development may simultaneously modify surface chemistry, roughness, and spatial heterogeneity, thereby affecting contact-line pinning and potentially changing the wetting regime. Since these contributions were not independently resolved, the CAH-derived parameters are interpreted as apparent descriptors of the overall wetting response rather than as intrinsic thermodynamic surface properties. In particular, the present measurements do not allow changes in surface chemistry to be distinguished quantitatively from morphological effects.

### 4.3. Exploratory Relationships Between Surface Wettability and Lake Water Characteristics

The relationships between lake water characteristics (Table 1) and the wettability parameters of the natural substratum (*Potamogeton lucens*, Table 3) were examined using young and old leaves collected from three freshwater lakes. The analysis revealed several potentially interesting relationships that may provide directions for further investigation of surface wettability in environmental assessment. In particular, the observable advancing contact angle, *θ_A_*, exhibited a similar tendency with Secchi depth in both leaf-age groups. Water transparency is often associated with phytoplankton biomass, as reflected by chlorophyll a concentration, although suspended mineral particles and colored dissolved organic matter may also contribute to light attenuation [39]. Thus, the observed ordering indicates an exploratory relationship between the advancing contact angle and water transparency that warrants investigation in a larger set of lakes. Interestingly, the same ordering of lakes was observed for young and old leaves, despite the pronounced differences in their absolute contact angles. However, the small differences between some lakes and the limited number of independent observations require further verification.

Another interesting relationship was found between the ammonium concentration in lake water and the age-associated changes in leaf-surface wettability. The difference in advancing contact angle between old and young leaves, defined as *Δθ_A_ = θ_A,old_ − θ_A,young_*, changed from −10.8° in Dabrowskie Lake to −10.4° in Zurominskie Lake and −9.9° in Lubowisko Lake. This corresponded to the same rank ordering of *Δθ_A_* and mean ammonium concentration across the three lakes. A similar ordering was observed for total nitrogen, dissolved oxygen, and TSI(Chl), indicating that the individual contributions of these environmental factors cannot be distinguished. Nevertheless, the ammonium relationship deserves particular attention in view of the findings of Ochieng et al. [40], who demonstrated different responses of young and mature *P. lucens* leaves to ammonium enrichment and identified glutamate dehydrogenase as an important component of ammonium detoxification in young leaves. Although the ammonium concentrations investigated in that study were considerably higher than those recorded in the present lakes, these findings provide a biological rationale for further examining whether nitrogen-related environmental conditions may influence age-associated changes in leaf-surface properties.

Other lake water characteristics also revealed potentially useful relationships with the wettability parameters. In particular, the difference in the receding contact angle between old and young leaves showed a corresponding rank ordering with water conductivity, whereas the corresponding differences in CAH and film pressure, *Π*, exhibited inverse rank ordering. This tendency was largely associated with Zurominskie Lake, where the receding contact angle changed only slightly between the two age groups, in contrast to the larger changes observed in the remaining lakes. These observations suggest that different components of the wetting response may reflect distinct aspects of the aquatic environment. The possible roles of water ionic composition, adsorption of natural surface-active compounds, epiphytic colonization, and mineral deposition on leaf surfaces therefore deserve further investigation.

The potential environmental significance of the observed relationships may also be considered in connection with biofilm accumulation on artificial substrata, as discussed in Section 4.2. The measured biofilm wet mass depended on sampler material, configuration, and exposure time, indicating that organic matter accumulation is a complex process that cannot be predicted from the advancing contact angle alone. Nevertheless, adsorption of dissolved organic compounds, epiphytic colonization, and subsequent biofilm development provide possible pathways through which environmental conditions may modify the outermost surface of submerged macrophytes and, consequently, their wettability. Direct measurements of biofilm accumulation on the investigated leaves were not available, and this mechanism remains to be verified. Although the present analysis was limited to three independent lakes and the observed relationships should be regarded as preliminary, the results identify promising directions for further research. In particular, studies covering a broader range of freshwater environments, with simultaneous measurements of water chemistry, leaf age, surface wettability, and biofilm accumulation, are needed to establish whether selected wettability parameters can serve as reliable indicators of environmental conditions and the state of submerged biological surfaces.

## 5. Conclusions

The captive bubble method, combined with axisymmetric bubble shape analysis (ABSA-P), provides a practical approach for characterizing the wettability of fully hydrated artificial and natural substrata submerged in freshwater environments. The outermost surface evolution of submerged biological systems—epilithon, epixylon, epipsammon, and epiphylon—can be monitored in situ at different stages of biofilm formation, from the development of a conditioning film to macrofouling. The wettability of submerged artificial substrata was found to vary with material type and sampler configuration. The exposed glass and PVC surfaces exhibited different combinations of contact angle hysteresis and apparent energetic parameters, demonstrating that their wetting behavior cannot be represented by a universal hydrophobization trend. Since the reference and exposed-surface datasets were obtained under different experimental conditions, the contribution of biofilm formation to these differences cannot be isolated from the present measurements alone.

The wettability measurements of *Potamogeton lucens* leaves revealed consistent differences between young and old leaves in all three investigated lakes. Older leaves exhibited lower advancing contact angles and higher apparent surface free energies and works of adhesion. These observations extended previously published reference measurements [11] and indicated reproducible age-associated differences in macrophyte surface wettability. However, the contributions of intrinsic leaf aging, surface erosion, adsorption of natural compounds, and epiphytic colonization could not be distinguished quantitatively.

Exploratory analysis identified several potentially interesting relationships between lake water characteristics and macrophyte surface wettability. In particular, the advancing contact angle exhibited a consistent relationship with Secchi depth in both leaf-age groups, supporting further investigation of its relationship with water transparency and associated optical conditions. Age-associated changes in the advancing contact angle also showed a possible relationship with ammonium concentration, whereas differences in the receding contact angle and CAH-derived parameters varied with water conductivity. These observations identify specific parameters for further investigation rather than establish validated environmental indicators.

The present results demonstrate the potential of surface wettability measurements for comparative characterization of submerged biological surfaces and for investigating their relationships with freshwater environmental conditions. Further studies involving a larger number of independent lakes, additional macrophyte species, and simultaneous measurements of water chemistry, surface wettability, and biofilm accumulation are required to clarify the underlying mechanisms and evaluate the applicability of this approach to environmental monitoring.

## Figures and Tables

**Table 1:** Morphometric, physical and chemical characteristics of the lakes studied. Minimum–maximum ranges in parentheses represent temporal variability during 2024, whereas individual values refer to the August 2024 sampling campaign.

Variable	Rekowo	Lubowisko	Dabrowskie	Zurominskie
Surface area (ha)	59.2	37.1	64.3	10.1
Max. depth (m)	11.8	7.6	15.2	5.0
Mean depth (m)	5.6	3.4	6.4	2.3
pH	8.0	7.3	7.8	7.7
Dissolved O_2_ (mg dm^−3^)	7.7	8.0	4.1	7.6
Phosphate PO_4_^3−^ (mg dm^−3^)	(0.006–0.038)	not detected	(0.022–0.025)	not detected
Nitrite NO_2_^−^ (mg dm^−3^)	(0.007–0.021)	(0.008–0.015)	(0.008–0.015)	(0.006–0.010)
Ammonium NH_4_^+^ (mg dm^−3^)	(0.027–0.048)	(0.035–0.175)	(0.01–0.039)	(0.026–0.143)
Secchi Depth (m)	1.5	2.1	2.2	1.8
Conductivity (μS cm^−1^)	320	140	190	360
Total P (mg dm^−3^)	(0.015–0.047)	(0.013–0.025)	(0.025–0.034)	(0.017–0.061)
Total N (mg dm^−3^)	(0.37–1.05)	(0.52–1.57)	(0.36–1.00)	(0.67–1.28)
TSI_SD_	54.2	49.3	48.6	51.5
TSI_Chl_	61.2	59.7	55.4	58.7
TSI_Ptot_	51.7	45.8	51.1	49.7

**Table 2:** Wettability parameters of artificial substrata measured using a common aqueous probe liquid (*γ_LV_* = 71.9 ± 0.7 mN m^−1^ at T = 22 °C), together with previously published reference data from [11].

Substratum	*θ_A_*	*θ_R_*	CAH	*Π*	*γ_SV_*	*W_A_*	*W_S_*	$\gamma_{\mathrm{SV}}^{d}$/*γ_SV_*
	(Deg)			(mN m^−1^)	(mJ m^−2^)			-
Polymeric								
1. PE	105.0	54.1	50.9 ± 1.4	60.7 ± 1.7	17.0 ± 0.7	53.3 ± 1.3	−90.5 ± 1.5	0.582 ± 0.006
2. PP	94	79	15.0 ± 1.4	18.7 ± 1.8	29.3 ± 0.9	66.9 ± 1.4	−76.9 ± 1.5	0.530 ± 0.006
3. PET	83.5	53.5	30.0 ± 1.4	34.6 ± 1.6	32.9 ± 0.9	80.0 ± 1.5	−63.7 ± 1.4	0.677 ± 0.006
4. PVDF (non-poled)	92	52	40.0 ± 1.4	46.8 ± 1.7	25.9 ± 0.8	69.4 ± 1.4	−74.4 ± 1.5	0.645 ± 0.006
5. PVDF (poled)	83.5	51	32.5 ± 1.4	37.1 ± 1.6	32.5 ± 0.9	80.0 ± 1.5	−63.7 ± 1.4	0.686 ± 0.006
6. PSU	86.5	45	41.5 ± 1.4	46.4 ± 1.6	29.2 ± 0.8	76.3 ± 1.5	−67.5 ± 1.4	0.692 ± 0.005
7. PMMA	85.1	67.5	17.6 ± 1.4	21.4 ± 1.7	34.3 ± 1.0	78.0 ± 1.5	−65.7 ± 1.4	0.617 ± 0.006
8. PTFE	106.8	79.3	27.5 ± 1.4	34.1 ± 1.8	19.2 ± 0.8	51.1 ± 1.3	−92.6 ± 1.5	0.474 ± 0.006
9. PVC	77.4	55.9	21.5 ± 1.4	24.6 ± 1.6	38.4 ± 0.9	87.6 ± 1.5	−56.2 ± 1.4	0.695 ± 0.006
Metallic								
10. Stainless steel	80.7	52.9	27.8 ± 1.4	31.7 ± 1.6	35.1 ± 0.9	83.5 ± 1.5	−60.3 ± 1.4	0.691 ± 0.006
11. Aluminum	81.9	68.9	13.0 ± 1.4	15.7 ± 1.7	37.4 ± 1.0	82.0 ± 1.5	−61.7 ± 1.4	0.625 ± 0.006
Other								
12. Wax-covered glass	101.6	91.2	10.4 ± 1.4	12.9 ± 1.8	25.8 ± 0.9	57.4 ± 1.4	−86.3 ± 1.5	0.444 ± 0.006
13. Bare glass [11]	50.2 ± 1.8	11.5 ± 1.5	38.7 ± 3.7	24.9 ± 2.6	54.5 ± 4.9	120.2 ± 9.8	−26.4 ± 4.2	0.905 ± 0.08
14. Rubber	75.0	47.5	27.5 ± 1.4	30.0 ± 1.6	38.8 ± 0.9	90.5 ± 1.5	−53.3 ± 1.3	0.734 ± 0.005
15. Gelatinous layer	82.7	77.9	4.8 ± 1.4	5.9 ± 1.7	39.1 ± 1.0	81.0 ± 1.5	−62.7 ± 1.4	0.584 ± 0.006
Natural								
16. *Potamogeton lucens* leaves [11]	73.4 ± 2.1	65.1 ± 1.8	8.3 ± 1.3	9.81 ± 0.81	44.3 ± 3.5	93.2 ± 6.8	−51.8 ± 4.9	0.69 ± 0.09

Previously published contact angle data: bare glass (row 13) and *Potamogeton lucens* leaves (row 16) were from [11].

**Table 3:** Wettability parameters and biofilm wet mass (BWM in mg cm^−2^, accumulated on the artificial samplers after 19 (index: ^1^) and 46 (^2^) days of deployment) of artificial samplers exposed in Rekowo Lake in August 2024, together with reference wettability data for *Potamogeton lucens* leaves from different locations. The artificial samplers’ wettability parameters were calculated using probe water with *γ_LV_* = 73.4 mN m^−1^.

Substratum/	*θ_A_*	*θ_R_*	CAH	*Π*	*γ_SV_*	*W_A_*	*W_S_*	$\gamma_{\mathrm{SV}}^{d}$/*γ_SV_*	BWM ^1^	BWM ^2^
Sampler	(Deg)			(mN m^−1^)	(mJ m^−2^)			-	(mg cm^−2^)	
Artificial										
stick-like samplers										
1. glass plate	39.1	35.4	3.7 ± 1.4	2.9 ± 1.1	64.5 ± 0.6	130.4 ± 0.8	−16.4 ± 0.8	0.898 ± 0.004	0.30	2.24
2. PVC foil (O)	57.8	45.7	12.1 ± 1.4	12.2 ± 1.4	53.4 ± 0.8	112.5 ± 1.1	−34.3 ± 1.1	0.808 ± 0.005	0.38	8.08
3. PVC foil (B)	53.7	42.7	11.0 ± 1.4	10.5 ± 1.3	55.9 ± 0.8	116.9 ± 1.0	−29.9 ± 1.0	0.832 ± 0.005	0.06	4.24
float-type samplers										
4. glass plate	45.7	41.6	4.1 ± 1.4	3.6 ± 1.3	61.4 ± 0.7	124.7 ± 0.9	−22.1 ± 0.9	0.862 ± 0.004	6.22	3.01
5. PVC foil (O)	34.9	28.4	6.5 ± 1.4	4.4 ± 1.0	65.7 ± 0.6	133.6 ± 0.8	−13.2 ± 0.7	0.925 ± 0.003	6.56	5.44
6. PVC foil (B)	42.6	33.1	9.5 ± 1.4	7.5 ± 1.1	61.9 ± 0.7	127.4 ± 0.9	−19.4 ± 0.9	0.894 ± 0.004	3.18	1.39
Natural										
7. young leaf [11]	73.4 ± 2.1	65.1 ± 1.8	8.3 ± 1.3	9.81 ± 0.81	44.3 ± 3.5	93.2 ± 6.8	−51.8 ± 4.9	0.69 ± 0.09	-	-
8. 1-year-old leaf [11]	63.4 ± 1.7	50.3 ± 3.2	13.1 ± 2.4	13.9 ± 1.9	49.2 ± 4.2	105.0 ± 7.6	−40.0 ± 5.2	0.77 ± 0.09	-	-
9. young leaf Dabrowskie lake	72.9	64.1	8.8 ± 1.4	10.2 ± 1.6	43.8 ± 0.9	92.52 ± 1.2	−50.5 ± 1.2	0.683 ± 0.006	-	-
10. 1-year-old leaf Dabrowskie lake	62.1	52.2	9.9 ± 1.4	10.3 ± 1.5	50.0 ± 0.8	105.0 ± 1.1	−38.0 ± 1.1	0.770 ± 0.005	-	-
11. young leaf Lubowisko lake	72.9	65.1	7.9 ± 1.4	9.3 ± 1.7	44.9 ± 0.9	94.2 ± 1.2	−51.4 ± 1.2	0.679 ± 0.006	-	-
12. 1-year-old leaf Lubowisko lake	63.1	51.4	11.6± 1.4	12.4 ± 1.5	50.0 ± 0.9	105.7 ± 1.2	−39.8 ± 1.2	0.769 ± 0.005	-	-
13. young leaf Zurominskie lake	73.6	59.0	14.6 ± 1.4	17.0 ± 1.6	42.8 ± 0.9	93.3 ± 1.2	−52.2 ± 1.2	0.699 ± 0.006	-	-
14. 1-year-old leaf Zurominskie lake	63.2	58.2	5.0 ± 1.4	5.5 ± 1.6	51.4 ± 0.9	105.5 ± 1.2	−40.1 ± 1.2	0.744 ± 0.005	-	-

Rows 1–6: artificial samplers deployed in Rekowo Lake on 11 August 2024. Rows 7–8: previously published data for current-year and one-year-old *Potamogeton lucens* leaves collected in Puck Bay on 14 October 2011 [11]. Rows 9–14: data for *Potamogeton lucens* collected at selected lakes, current experimental data run. BWM data were not available for the macrophyte leaves (rows 7–14), as indicated by dashes.

## Data Availability

The original contributions presented in this study are included in the article. Further inquiries can be directed to the corresponding author.
